# Subjective Health Literacy among School-Aged Children: First Evidence from Lithuania

**DOI:** 10.3390/ijerph16183397

**Published:** 2019-09-13

**Authors:** Saulius Sukys, Laima Trinkuniene, Ilona Tilindiene

**Affiliations:** Department of Physical and Social Education, Lithuanian Sports University, Sporto 6, LT-44221 Kaunas, Lithuania

**Keywords:** health literacy, school health promotion, adolescence

## Abstract

Health literacy as a set of competencies to promote and sustain health has received significant research attention, particularly in studies on adults. Improving health literacy at an early age is crucial to personal health and development, so there is a need to investigate the health literacy of school-aged children. The aims of this study were to determine the level of subjective health literacy among adolescents in Lithuania and to examine the association between health literacy, school achievement, health education in schools, and family affluence. Health literacy was assessed using a brief Health Literacy for School-Aged Children instrument on a representative sample of 2369 subjects (from the 7th to 10th grades). Overall, 12.1% of all respondents had low, 70.5% moderate, and 17.4% a high level of health literacy. School achievements were found to be a significant predictor of health literacy, as were the number of school-based health promotion events. Family affluence also predicted an increased level of health literacy. This study was the first nationally representative examination of this topic in Lithuania and it highlighted the alarming finding that less than one-fifth of adolescents had high health literacy.

## 1. Introduction

Although children and young people are considered to be healthier than adults, attention to their health is important because behavioral patterns that help determine current health status and future health outcomes are emerging at this age [1]. This stage of life, particularly adolescence, is significant because empirical evidence suggests an increase in health complaints [2,3], a decline in subjective well-being, and persistent negative health behaviors [1] over time and age. Therefore, the consideration of factors related to the health and education of children remains a relevant question for public healthcare services.

Although the health behavior of children is still a substantial concern, the manner in which people learn and make sense of health-related information is important [4]. From the perspective of health promotion, it is vital to analyze the concept of health literacy (HL) and children’s HL levels as well as educational perspectives.

The HL concept originally focused upon healthcare services. More recently, the meaning of this concept was expanded to be “the personal, cognitive and social skills which determine the ability of individuals to gain access to, understand, and use information to promote and maintain good health” [5] (p. 263). Moreover, three dimensions of HL were characterized: functional, interactive, and critical [5]. The latest systematic analyses on the HL of children and youth revealed that there are substantially different definitions and models of HL [6]. In general, HL comprises different health-related skills, competences, knowledge, and motivational components [7]. Emphasizing the importance of the environment in the development of HL [8], children’s HL should be seen as part of their learning outcomes [9]. Because children spend a lot of time at school, HL as a learning outcome at school includes the range of knowledge and competencies (as partly overlapping components) that can empower them to understand themselves, others, and the world, as well as to make sound health decisions [9]. Those components include theoretical and practical knowledge, critical thinking, self-awareness, and a notion of citizenship that collectively require not only understanding health-related information but also being active while taking responsible actions to promote one’s own and others’ health. Hence, HL as a construct enables children—as future adults—to develop their knowledge and participate fully in their community and in wider society [10]. Therefore, it is essential to nurture HL as early as possible to contribute to the health and development of children and adolescents [11].

Although the focus on HL in children and adolescents is increasing, there is more research being performed on adult HL. Although studies on HL in children have increased in recent years [6,12], childhood and adolescent HL is still underresearched [13]. It should be noted that, in contrast to research on adults, studies on children should consider such distinguishing factors as developmental changes, dependence on parents and peers, unique patterns of health, and demographic patterns [14]. Thus, research on children and adolescents suggests that insufficient or low HL is associated with various adverse health outcomes, such as smoking [15], alcohol use [16], and obesity [17]. Moreover, an analysis of previous research reveals that lower HL is linked to less engagement in health-promoting behaviors [12], poorer self-assessed health [18], and lower quality of life [19]. However, other findings suggest that higher HL is linked positively to better health outcomes [20]. Focusing on school-aged children, a positive relationship has been demonstrated between HL and school achievements as well as learning aspirations [21].

Evidence suggests that there are health disparities between the health outcomes of boys and girls [1]. Reviews of the research have shown that the data on comparing HL according to gender are quite controversial [12] (i.e., it cannot be asserted firmly whether girls or boys have higher HL). Previous studies have also revealed the links between the HL of children and their family’s socioeconomic status [18,21]. However, a cross-country comparative analysis has also shown that the links between HL and socioeconomic indicators vary from country to country [18], a finding that also encourages a deeper understanding of country-specific situations.

In Lithuania, only a few studies have been carried out so far on HL. These studied the functional literacy of patients in adult hospitals [22] and in primary healthcare centers [23]. The HL of university students has also been studied [24], but research regarding the HL of schoolchildren has yet to be performed. The lack of such analyses might be related to not having specific government policies on HL [25]. Therefore, it is relevant to investigate schoolchildren’s HL in Lithuania, especially as the national health behavior outcomes are compared with other European countries [1]. In this regard, since 2012, the Health Education General Program of the Minister for Education and Science has been approved in Lithuania (entitled the “Health and Sexual Education and Training Program for the Family” since 2016). This program foresees the integration of health education into the educational process to help schoolchildren gain a holistic health concept, develop health-related abilities, habits and attitudes, take responsibility for their own and others’ health, and to encourage them to choose a healthy lifestyle.

The main aim of this study was to determine the level of subjective HL among adolescents in Lithuania. The study also sought to examine the associations between HL, school achievement, health education in schools, and family affluence.

## 2. Materials and Methods

### 2.1. Participants

The population selected for sampling included 7th-, 8th-, 9th- and 10th-grade children attending general schools in Lithuania. Participants were selected using a clustered hierarchical sampling design, where the initial sampling unit was the school class. The sample size for each of four grades was calculated assuming a 95% confidence level and ±3% confidence interval. Assuming the total number of children within each grade, the recommended sample size for each group was no less than 583 children. First, schools from different counties of Lithuania were selected. Then, one class from each of the 7th to 10th grades was selected randomly at each of the schools. In total, 76 classes from 29 schools from the entire country were recruited to ensure the required number of surveyed students. Participation in the study was voluntary and anonymous, and respondents were not offered any incentives to participate. The survey included 2489 students. After the screening, we excluded respondents who failed to report data on gender, all items on the Health Literacy for School-Aged Children, and those participants who provided several unlikely responses throughout the questionnaire. This strategy provided a final sample of 2369 adolescents (47.5% boys) aged 13–16 years (mean age 14.44, standard deviation, SD ± 1.14).

### 2.2. Measures

#### 2.2.1. Health Literacy

The subjects’ subjective HL was measured using a brief Health Literacy for School-Aged Children (HLSAC) instrument [26]. Before the survey, HLSAC was translated into Lithuanian using a forward and backward translation procedure. It was first translated by three independent translators from the English version into Lithuanian. Next, two authors of this paper discussed any disagreement in the translation until they reached agreement. Then, the scale text was translated backward from Lithuanian to English by two translators. To test the understanding of the Lithuanian HLSAC, we administered the instrument to 40 adolescents, who confirmed the clarity of the scale. This 10-item instrument contains two items from each of the five core components of HL: theoretical knowledge, practical knowledge, critical thinking, self-awareness, and citizenship. Participants responded to the stem postulate, “I am confident that ...” using a 4-point Likert-type scale ranging from “not at all true” to “absolutely true”. In assessing HL levels, the sum scores for all items were calculated. Then, three HL levels were defined as: “low” (score 10–25), “moderate” (score 26–35), or “high” (score 36–40) [21]. The original HLSAC instrument had high internal consistency (Cronbach’s α = 0.93). In this study, Cronbach’s α value was 0.88.

#### 2.2.2. School Achievement

Participants were asked to indicate their school achievement in their final term using the following question: “In my latest term my mark was […]”. In Lithuanian children, school achievement is evaluated from 1 to 10. In this study sample, responses (marks) ranged from 4 to 10. The marks thus obtained were grouped to form three categories (marks 4–6, 7–8, and 9–10).

#### 2.2.3. Perceived School Focus on Health Education

Health education in this study was assessed as the perceived school focus on a healthy lifestyle. First, we measured adolescents’ opinion regarding some particular healthy lifestyle promotion activities. Specifically, participants were asked: “Does your school pay enough attention to the basics of a healthy lifestyle?” [27] with six items: physical activity, nutrition, smoking prevention, alcohol use prevention, drug use prevention, and prevention of bullying. All items were answered on a 4-point Likert-type scale: “do not know” = 0, “too little” = 1, “enough” = 2 and “too much” = 3. Second, the frequency of school-based health promotion events as additional variable was assessed. Specifically, participants were asked “Has the school held events for schoolchildren’s health promotion in the last three to four months?” with five options: “do not know” = 0; “no” = 1; “was held, but earlier” = 2; “was held once” = 3; and “yes, more than once” = 4. Analyzing the data, perceived school focuses on physical activity, nutrition, as well as alcohol, smoking and drug use, prevention of bullying were used as predicting variables for HL. Additionally, the frequency of school-based health promotion events was also used as a predicting variable for HL.

#### 2.2.4. Family Affluence

The Family Affluence Scale [28] was used for this study. This scale includes six items that are associated with parental income and hence function as measures of adolescents’ socioeconomic status. The questions included items about: occupancy of bedrooms, dishwasher ownership, ownership of a car, number of computers, number of bathrooms, and holidays abroad. The response options for the first two questions were “No” = 0 and “Yes” = 1. For the third question, response options were “No” = 0, “Yes, one” = 1, and “Yes, two” = 2. For the remaining questions, responses were “No” = 0, “Yes, one” = 1, “Yes, two” = 2, and “More than two” = 3. The respondents were divided into three affluence groups: low (sum scores 0–6), medium (sum scores 7–9), and high (sum scores 10–13).

### 2.3. Ethical Approval

All participants gave their informed consent for inclusion before they participated in the study. The study was conducted in accordance with the Declaration of Helsinki, and the research protocol was approved by the Research Ethics Committee of Lithuanian Sports University (NR. SMTEK-28).

### 2.4. Data Analysis

We analyzed data using IBM SPSS Statistics (version 25; IBM Corp., Armonk, NY, USA). All statistical analyses were conducted for the total sample, separately for boys and girls, and for the 7th to 10th grades. Cronbach’s α values were calculated to evaluate internal consistency. The descriptive statistics for HL included means, SDs, and percentage distributions of HL levels and predictor variables. A two-way analysis of variance (ANOVA) was used to compare differences between the group means (for gender and grade), and gender and grade interaction effects on HL. In all instances (comparing more than two groups) where the null hypothesis was rejected, a post-hoc Tukey’s test was computed. Chi-square tests were used to compare the distribution of percentages between groups. Pearson’s correlation coefficients were calculated to examine the interrelationship between HL and other predictor variables. Multiple regression analyses were used to measure the relationship between HL and school achievement and perceived school focus on healthy lifestyle promotion at school. For all statistical analysis, we set statistical significance at *p* < 0.05.

## 3. Results

The overall mean HL score was found to be 30.87 among the entire sample (Table 1). The lowest subjective HL was found among the 7th-grade boys (mean score 29.74), and the highest subjective HL was determined for the 9th-grade girls (mean score 32.17). A two-way ANOVA was conducted to examine the differences between gender and grade. Analysis showed that the HL mean score for girls was significantly higher than for boys (*F*_1,2361_ = 23.47, *p <* 0.001, *η*^2^ = 0.01). Comparing HL mean scores between grades, significant differences were found (*F*_3,2361_ = 10.84, *p <* 0.001, *η*^2^ = 0.02). Tukey’s test revealed that 9th- and 10th-grade schoolchildren scored statistically significantly higher than those in the 7th and 8th grades (*p* < 0.001). The results did not reveal significant gender or grade interaction effects on HL mean scores (*F*_3,2361_ = 1.23).

The participants’ HL was categorized into three levels. Among the entire sample, 12.1% had low HL, 70.5% had moderate HL and 17.4% achieved a high level of HL (Table 1). Comparison by gender showed that there were significantly fewer girls than boys with lower HL (χ^2^ (2, *N* = 2369) = 21.48, *p* < 0.001). In addition, the proportion of boys with a low level of HL was higher in all grades. The proportion of subjects with a high level of HL was highest in the 9th grade, and the lowest was in the 7th grade.

The HL instrument used contains ten items related to theoretical and practical knowledge, critical thinking, self-awareness, and citizenship. Percentage distribution on every item showed that approximately one in four children had difficulties related with critical thinking and around 20% reported having difficulties regarding citizenship (response options: “not at all true” or “barely true”; Supplementary Table S1). Fewer difficulties were reported regarding theoretical and practical knowledge (~15%). Boys reported more difficulties than girls on all HL items.

The descriptive statistics for the predictors (Table 2) showed that better school achievement results were found among girls than boys. More girls also reported sufficient school focus on a healthy lifestyle, except for school efforts related to physical activity. In addition, there was no difference in the responses of girls and boys regarding school-based health promotion events in the previous three months. Regarding grade, older pupils more often reported sufficient school focus on smoking, alcohol or drug use, and prevention of bullying. Participants from the 9th and 10th grades also more often reported school-based health promotion events held in the previous three months.

Next, correlation analyses between HL and other predictor variables were executed. Results revealed a positive significant correlation between HL and school achievements (Pearson’s r = 0.26). Positive correlations between HL and perceived school focus on a healthy lifestyle (physical activity, nutrition, smoking, alcohol or drug use, and prevention of bullying) and the frequency of school-based health promotion events varied between r = 0.06 and r = 0.12. Better family affluence was also positively correlated with HL (r = 0.12).

Finally, regression analyses were conducted to investigate the relationship between HL and predictors. The predictive variables entered were: school achievement, perceived school focus on healthy lifestyle promotion activities (separate for physical activity, nutrition, smoking, alcohol or drug use, and the prevention of bullying), and the frequency of school-based health promotion events. Gender, grade, and family affluence were used as controlling variables (Table 3). Analysis demonstrated that gender, grade, and family affluence were significant predictors of HL. School achievement was also significant predictor of HL. Among variables related to a perceived school focus on healthy lifestyle promotion, only the prevention of bullying was a significant predictor of HL. School-based health promotion events held in the previous three months were also a significant predictor of HL.

## 4. Discussion

Given the importance of promoting HL among children and adolescents and in light of limited research related to HL in some countries, the main aim of this study was to determine the level of subjective HL among adolescents in Lithuania. In addition, we sought to examine the associations between HL, school achievement, health education in schools, and family affluence. We found that less than one-fifth of adolescents had high HL levels and about one-tenth of adolescents had low levels. Research in the USA has demonstrated that about 10% of adolescents have limited or low HL [29,30]. Similar studies in different Asian countries have revealed that 10%–20% of adolescents have limited or low HL [31,32,33]. On the other hand, the studies mentioned above examined functional HL, whereas when analyzing HL as a broader construct, the data for adolescents differ slightly. Research on 15–29-year-olds in Germany found that 6.8% of respondents had inadequate HL and 40.5% had problematic HL [34]. Moreover, 11.3% of adolescents in the Czech Republic had inadequate HL and 47.3% had adequate HL [35]. A recent study in Finland showed that approximately one-tenth of respondents had low HL [21], similar to our findings. However, the number of adolescents surveyed in our study having a high level of HL was half that reported previously.

Comparing HL levels by gender, we found that the level was higher in girls than boys. Recent studies of Finnish adolescents found a similar distribution [21]. These data are partly unexpected because in Lithuania, as in many other countries, girls self-evaluate their health as being worse than boys [1]. However, the better HL data for girls are probably determined by their better higher learning motivation [36] and cultural, school environment, and education peculiarities [37].

These findings on HL in adolescents can be interpreted by referring to the practice of schoolchildren’s health education in Lithuania. The country has an approved “Health, Sexual and Family Education” program, which covers the fields of health, healthy lifestyle, physical, mental, and social health, and their development. However, this program foresees an integrated application of health education (i.e., each school foresees how health education will be integrated into the content of subjects and implemented through nonformal education activities). Therefore, health education is not an independent compulsory subject in the curriculum for secondary schools. Although integrated education can be meaningful in the education process, it also presents some challenges. Previous studies have found that teachers perceive their responsibility and their role in implementing integrated health education differently [38]. Some teachers comprehend this area as being the responsibility of every teacher, others consider it to be a collaboration between teachers and parents, and some teachers are skeptical about integrating health education into individual subjects. In fact, challenges with integrated health education have also been documented in other countries [39]. The above-listed challenges can lead to inadequate education in HL for children. This finding is partly confirmed by surveys, with children claiming that they do not have sufficient information regarding healthy lifestyles [40].

Health education at school is a complex process whereby key health messages are presented via the curriculum and other school-based practices. Our research disclosed a positive relationship between organized activities for healthy lifestyle promotion and children’s HL. Interestingly, among different activities focusing on healthy lifestyle promotion, prevention of bullying activities alone was found to be positively linked to children’s HL. It should be noted that, according to the Health Behaviour in School-Aged Children (HBSC) study, bullying among schoolchildren in Lithuania appears to be the issue most in common with other countries [1]. As bullying is known to be linked to various health complaints, poorer self-rated health [41], and also lower understanding of health-related quality of life [42], this issue has received a significant amount of attention in our country. A variety of prevention programs have been implemented in recent years to address this issue such as the bullying prevention programs (e.g., the Olweus bullying prevention program) found in most schools. Evidence suggests that school-based bullying prevention programs focus on developing social skills, improving such competences as self-management, self-awareness, and self-regulation, establishing positive relationships [43], and creating a school climate that reduces involvement in harmful behavior [44], which contributes positively to mental health [45] and mental HL [46]. It can be assumed that the aims of such programs correspond with knowledge and competences related to HL. Specifically, in the current study, HL was assessed not only as theoretical, practical knowledge and critical thinking related to health information but also as self-awareness and citizenship, which help the children to know themselves and other people better. Consequently, the established relationship between perceived prevention of bullying and HL has practical novelty, suggesting that targeting to prevent bullying can also increase children’s HL.

Here we found that schoolchildren with higher achievement levels also had increased levels of HL. This finding is consistent with previous studies suggesting that school achievement is a predictive variable for adolescents’ HL [21,47]. Another study has also suggested that lower learning achievement is linked not only with lower HL but also with poorer health outcomes [20]. Because learning outcomes are the result of an educational process, this issue raises questions related to schooling activities in general, and specifically for health promotion. Analyzing these questions, it is important to focus on the lower socioeconomic status of a family because it is also associated with poorer school achievement [20] and with lower HL [18,20,21]. In our study, family affluence level also predicted a higher level of HL. Nevertheless, we found that almost 40% of the families in our study were categorized as having low affluence. Therefore, it is important to study the moderating role of a family’s socioeconomic status in the relationship between children’s HL and health behavior.

This study had some limitations. The research design was cross-sectional, and the results only describe differences between samples from the 7th to 10th grades. Moreover, the amount of explained variance remained low in the regression model explaining HL, and this encourages further studies. For instance, a longitudinal study could provide further evidence regarding the development of HL. In addition, school activities related to health promotion were reported by the children themselves, who represent a population that may have issues with respect to the retention of information. It would also be useful to use school document analyses that collect information about health promotion activities and the content of such activities.

## 5. Conclusions

This was the first nationally representative examination of adolescents’ subjective HL in Lithuania, applying a specifically developed instrument for the target group. The present study highlighted the alarmingly low level of HL because less than one-fifth of adolescents had high HL. We also found that higher HL was associated with higher school achievement and that the number of school activities was related to health promotion. An important message of our study is that school bullying prevention programs are positively related to children’s HL. The findings revealed a link between higher family affluence and improved HL. Overall, our results demonstrate the importance of understanding the HL of Lithuanian adolescents as well as the requirement for HL education in school-based education systems.

## Figures and Tables

**Table 1:** Adolescents’ subjective health literacy: comparison by gender and age.

Grade Groups	Mean	SD	*α*	Levels of Health Literacy %		
				Low HL	Moderate HL	High HL
7th grade	30.19	5.15	0.87	16.5	69.3	14.2
Girls	30.66	4.73		13.1	73.4	13.5
Boys	29.74	5.50		19.9	65.3	14.8
8th grade	30.25	5.35	0.89	14.3	71.3	14.4
Girls	30.46	4.64		11.7	76.9	11.4
Boys	30.04	5.97		16.8	65.8	17.4
9th grade	31.57	5.06	0.89	8.5	70.1	21.4
Girls	32.17	4.84		6.0	69.1	24.9
Boys	30.88	5.23		11.3	71.4	17.3
10th grade	31.41	5.09	0.88	9.4	71.1	19.5
Girls	32.05	4.43		6.6	73.2	20.2
Boys	30.57	5.74		13.0	68.4	18.6
Total	30.87	5.20	0.88	12.1	70.5	17.4
Girls	31.39	4.72	0.87	9.2	73.0	17.8
Boys	30.29	5.62	0.90	15.4	67.6	17.0

**Table 2:** Adolescents’ school achievement, perceived school focus on health and family affluence.

Variables	Total	Gender		χ^2^ (df)	Grades				χ^2^ (df)
	*n* = 2369	Girl	Boy		7th Grade	8th Grade	9th Grade	10th Grade	
		*n* = 1244	*n* = 1125		*n* = 586	*n* = 582	*n* = 616	*n* = 585	
	*n* (%)	*n* (%)	*n* (%)		*n* (%)	*n* (%)	*n* (%)	*n* (%)	
**School achievement**									
Marks 4–6	385 (16.3)	118 (9.5)	267 (23.7)	150.7 (2) ***	112 (19.1)	99 (17.1)	84 (13.7)	90 (15.4)	9.63 (3)
Marks 7–8	1234 (52.1)	615 (49.4)	619 (55.0)		301 (51.4)	286 (49.1)	336 (54.5)	311 (53.2)	
Marks 9–10	750 (31.6)	511 (41.1)	239 (21.3)		173 (29.5)	197 (33.8)	196 (31.8)	184 (31.5)	
**Perceived school focus on** ^a^									
Physical activity	1789 (75.5)	962 (77.3)	827 (73.5)	6.49 (3)	455 (77.6)	446 (76.6)	456 (74.0)	432 (73.8)	5.28 (9)
Nutrition	1240 (52.3)	643 (51.7)	597 (53.1)	11.21 (3) **	325 (55.5)	326 (56.0)	306 (49.7) ^7,8^	283 (48.4) ^7,8^	29.15 (9) ***
Smoking prevention	1227 (51.8)	677 (54.4)	550 (48.9)	15.13 (3) **	275 (46.9)	305 (52.4)	318 (51.6) ^7,8^	329 (56.2) ^7,8^	52.8 (9) ***
Alcohol use prevention	1239 (52.3)	683 (54.9)	556 (49.4)	15.55 (3) **	269 (45.9)	304 (52.2) ^7^	326 (52.9) ^7^	340 (58.1) ^7,8,9^	70.17 (9) ***
Drug use prevention	1193 (50.4)	652 (52.4)	541 (48.1)	13.55 (3) **	254 (43.3)	283 (48.6)	320 (51.9) ^7^	336 (57.4) ^7,8,9^	66.7 (9) ***
Prevention of bullying	1329 (56.1)	726 (58.4)	603 (53.6)	20.97 (3) ***	314 (53.6)	308 (52.9)	359 (58.3)	348 (59.5) ^7,8^	20.91 (9) **
**Frequency of school-based health promotion events**									
Once or more times	1195 (50.5)	629 (50.6)	566 (50.4)	13.78 (4) **	282 (48.1)	246 (42.3) ^7^	328 (53.3) ^7,8^	339 (57.9) ^7,8^	102.8 (12) ***
**Family affluence**									
Low	898 (37.9)	511 (41.1)	387 (34.4)	13.64 (2) ***	219 (37.4)	224 (38.4)	221 (35.9)	234 (40.0)	4.66 (6)
Medium	975 (41.2)	500 (40.2)	475 (42.2)		248 (42.3)	243 (41.8)	250 (40.6)	234 (40.0)	
High	496 (20.9)	233 (18.7)	263 (23.4)		119 (20.3)	115 (19.8)	145 (23.5)	117 (20.0)	

Note: ^a^ The relative number of adolescents reported “enough” is presented. ^7,8,9^—significant difference from the grade indicated by the respective number (adjusted *p* Value—Benferroni method). ** *p* < 0.01, *** *p* < 0.001.

**Table 3:** Regression analysis predicting adolescents’ subjective health literacy.

Predictive Variables	B	*B* 95% CI	*β*	*t*	A*R*^2^
**Gender**	1.12	0.71< >1.53	0.11	5.32 ***	
**Grade**	0.47	0.29< >0.66	0.10	5.01 ***	
**Family affluence**	0.79	0.52< > 1.07	0.11	5.64 ***	
					0.04 ***
**School achievement ^a^**	1.77	1.46< >2.09	0.23	10.99 ***	
**Perceived school focus on**					
Physical activity	0.06	−0.38< >0.39	0.01	0.23	
Nutrition	0.11	−0.19< >0.41	0.02	0.72	
Smoking prevention	0.20	−0.62< >0.23	0.03	0.92	
Alcohol use prevention	0.14	−0.35< >0.63	0.02	0.57	
Drug use prevention	0.04	−0.37< >0.46	0.02	0.20	
Prevention of bullying	0.43	0.14< >0.72	0.07	2.91 **	
**School-based health promotion events**	0.27	0.13< >0.40	0.08	3.86 ***	
					0.09 ***

Note. A*R*^2^ = R^2^ unique to each step, ** *p* < 0.01, *** *p* < 0.001, CI = confidence interval, **^a^** all independent variables adjusted for gender, grade, and family affluence.

## Supplementary Materials

The following are available online at https://www.mdpi.com/1660-4601/16/18/3397/s1, Table S1. Percentage distributions of the items in the Health Literacy by gender.
