# Supplementary material for: Subjective Health Literacy among School-Aged Children: First Evidence from Lithuania
Source: Int J Environ Res Public Health. 2019 Sep 13;16(18):3397. doi: 10.3390/ijerph16183397 (PMC6765916; doi:10.3390/ijerph16183397)
Supplement: Supplementary file 1 [file ijerph-16-03397-s001.pdf]

Supplementary Table 1. Percentage distributions of the items in the Health Literacy by gender

	Boys (n=1125)				Girls (n=1244)				
	Not at all true	Barely true	Somewhat true	Absolutely true	Not at all true	Barely true	Somewhat true	Absolutely True	χ^2 (df)
Theoretical knowledge									
Having good information regarding health	3.4	8.8	57.8	30.0	1.7	7.6<br>1	62.5	28.2	10.8(3)**
Ability to give examples of things that promote health	4.9	18.8	50.8	25.6	1.7	7.0	52.1	29.2	22.92(3)***
Practical knowledge									
Ability to find health-related information that is easy to understand	3.7	15.0	47.2	34.1	1.4	10.2	46.9	41.5	31.55(3)***
Ability to follow the instructions given by doctors and nurses	4.7	11.8	39.5	44.0	2.2	8.1	37.3	52.4	28.85(3)***
Critical thinking									
Ability to decide if health-related information is right or wrong	3.9	24.5	49.6	22.0	2.7	19.0	56.2	22.1	15.53(3)***
Ability to compare health-related information from different sources	5.6	25.5	50.4	18.5	2.9	22.3	56.1	18.7	16.36(3)***
Self-awareness									
Ability to justify one´s own choices regarding health	4.1	16.9	54.8	24.2	1.4	14.5	54.2	29.9	26.65(3)***
Ability to judge how one´s own behaviour affects one's health	3.4	12.1	46.8	37.7	1.5	7.6	48.1	42.8	24.47(3)***
Citizenship									
Ability to judge how one's own actions affect the surrounding natural environment	4.0	18.3	49.2	28.5	1.8	13.2	54.3	30.7	22.96(3)***
Ability to give ideas on how to improve health in one's immediate surroundings	4.7	20.3	53.9	21.1	2.7	19.2	56.2	21.9	7.98(3)*

Note: * p < .5, ** p < .01, *** p < .001
